# New-Onset Diabetes Educator to Educate Children and Their Caregivers About Diabetes at the Time of Diagnosis: Usability Study

**DOI:** 10.2196/diabetes.9202

**Published:** 2018-06-06

**Authors:** Angelina Bernier, David Fedele, Yi Guo, Sarah Chavez, Megan D Smith, Jennifer Warnick, Leora Lieberman, François Modave

**Affiliations:** 1 Division of Endocrinology Department of Pediatrics University of Florida Gainesville, FL United States; 2 Department of Clinical & Health Psychology University of Florida Gainesville, FL United States; 3 Department of Health Outcomes and Biomedical Informatics University of Florida Gainesville, FL United States

**Keywords:** mHealth, information technology, diabetes education, pediatrics

## Abstract

**Background:**

Diabetes self-management education is essential at the time of diagnosis. We developed the New-Onset Diabetes Educator (NODE), an animation-based educational web application for type 1 diabetes mellitus patients.

**Objective:**

Our hypothesis is that NODE is a feasible, effective and user-friendly intervention in improving diabetes self-management education delivery to child/caregiver-dyads at the time of diagnosis.

**Methods:**

We used a pragmatic parallel randomized trial design. Dyads were recruited within 48 hours of diagnosis and randomized into a NODE-enhanced diabetes self-management education or a standard diabetes self-management education group. Dyads randomized in the NODE group received the intervention on an iPad before receiving the standard diabetes self-management education with a nurse educator. The Diabetes Knowledge Test 2 assessed disease-specific knowledge pre- and postintervention in both groups, and was compared using *t* tests. Usability of the NODE mobile health intervention was assessed in the NODE group.

**Results:**

We recruited 16 dyads (mean child age 10.75, SD 3.44). Mean Diabetes Knowledge Test 2 scores were 14.25 (SD 4.17) and 18.13 (SD 2.17) pre- and postintervention in the NODE group, and 15.50 (SD 2.67) and 17.38 (SD 2.26) in the standard diabetes self-management education group. The effect size was medium (Δ=0.56). Usability ratings of NODE were excellent.

**Conclusions:**

NODE is a feasible mobile health strategy for type 1 diabetes education. It has the potential to be an effective and scalable tool to enhance diabetes self-management education at time of diagnosis, and consequently, could lead to improved long-term clinical outcomes for patients living with the disease.

## Introduction

In the United States alone, close to 19,000 new pediatric cases of type 1 diabetes mellitus are diagnosed each year [1]. Living with type 1 diabetes requires drastic lifestyle changes both for the child and the caregiver. The delivery of diabetes self-management education and support (DSMES) at the time of diagnosis to children and families through in-person diabetes education and reading materials are integral components of new-onset diabetes self-management [2], [3]. Although standard of care DSMES is effective, evidence indicates that knowledge acquisition and retention could be substantially improved by using multimedia strategies (eg, images, videos) rather than text, particularly in the context of health education and literacy [4]. Thus, image delivery methods could improve long-term clinical outcomes and costs associated with lifetime diabetes management [5]. The goal of this pilot study is to present and assess the usability and preliminary efficacy of NODE, an animated educational web-based application designed to complement the standard of care in DSMES among a representative sample of newly diagnosed children with type 1 diabetes mellitus and their parent caregivers.

## Methods

**Figure 1:**
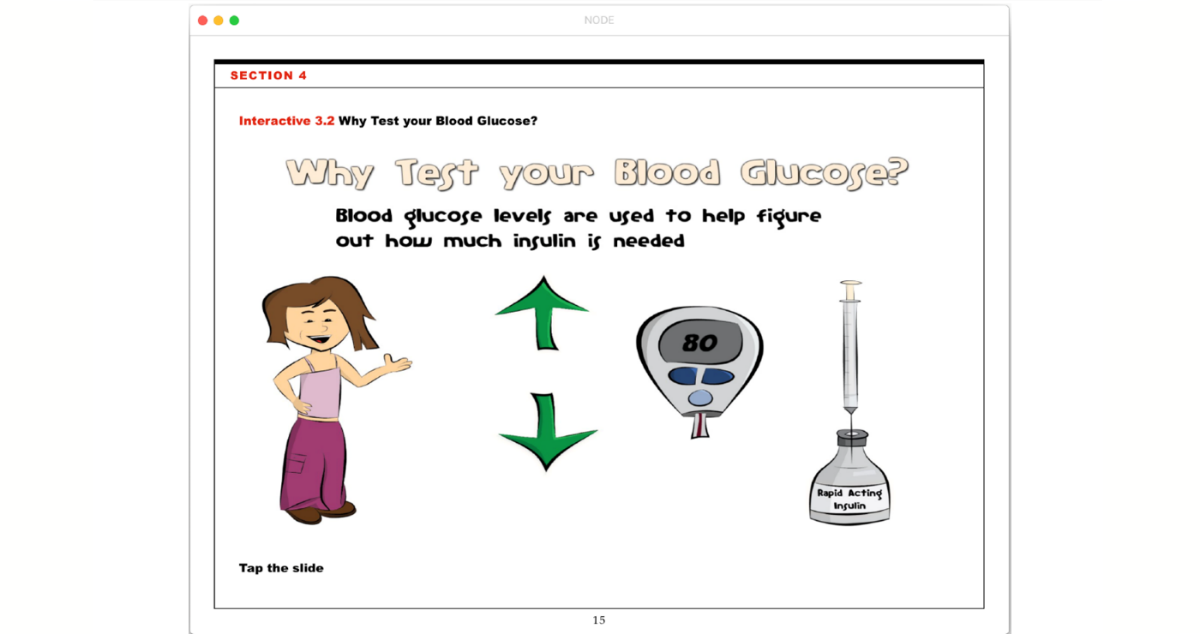
New-Onset Diabetes Educator (NODE). This page titled “Why Test your Blood Glucose?” from the Blood Glucose Monitoring module demonstrates interactivity as patients are prompted to tap the up and down arrows. Depending on their selection, the blood glucose value in the meter screen increase or decreases while simultaneously filling or emptying the syringe in the insulin vial. Children can directly see how higher blood glucose values require higher insulin doses.

**Figure 2:**
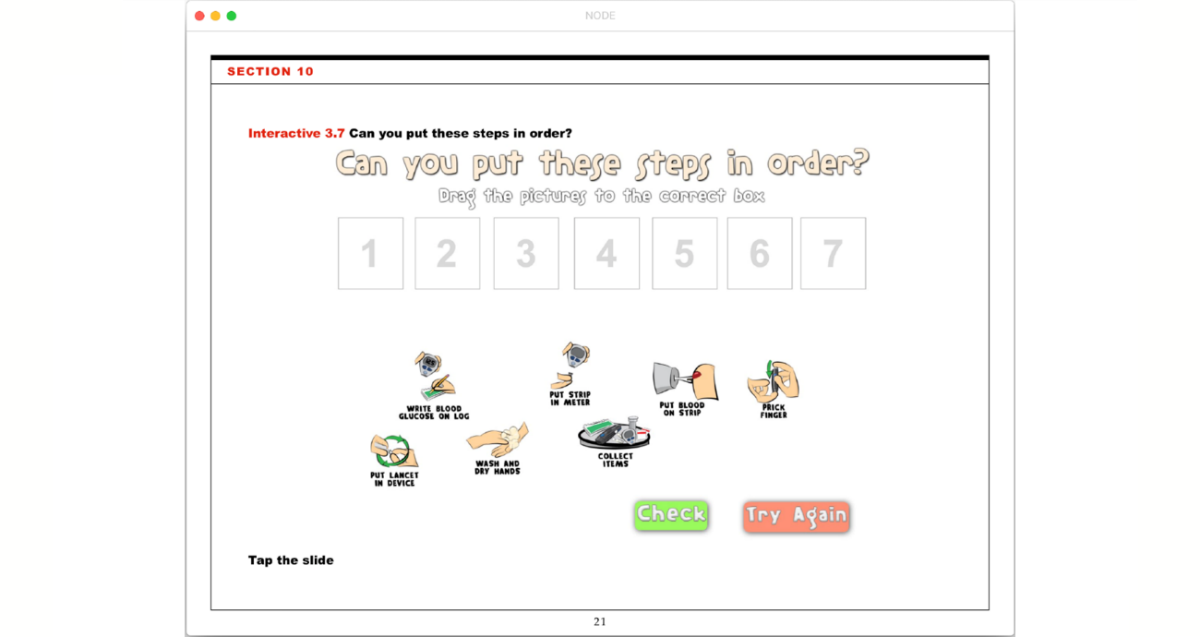
New-Onset Diabetes Educator (NODE) - Interactive Learning. This page titled “Can you put these steps in order?” is a game that tests a patient’s ability to drag the various steps needed for blood glucose monitoring into the correct order and check their results.

The NODE program was developed to run as an iBook on the standard Apple iOS mobile operating system to complement standard DSMES. Multiple disciplines were involved in the development of NODE content and presentation including pediatric endocrinologists, certified diabetes educators, and dietitians; in addition to artists, programmers, web designers and teachers. Each element and iteration were reviewed with patients and their families in clinical and camp settings to guide the look and usability. Following the outline and content of teaching educational materials common to new-onset education, eight modules were designed to cover basic diabetes self-management topics such as: What is Diabetes, Glucose Monitoring, Insulin, Hypoglycemia or Low, Hyperglycemia or High, Nutrition, Exercise and Diabetes, and Personal Management Plan*.* The presentation styles within these modules incorporate illustrations, interactive animations (see Figure 1), short cartoons, demonstration videos, and simple games (see Figure 2).

The purpose of NODE is to facilitate and foster diabetes knowledge acquisition and retention, aimed at expanding and enhancing standard DSMES materials and dissemination methods. The objectives for the current study were three-fold: 1) assess the usability of NODE; 2) monitor the feasibility of implementation; and 3) assess the preliminary efficacy of NODE in improving standard DSMES at diagnosis. The pilot study was approved by the Institutional Review Board, consent and assent were obtained prior to enrollment.

**Figure 3:**
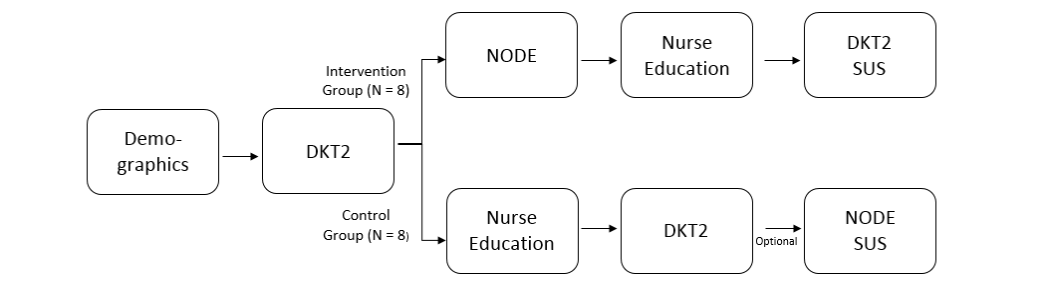
Intervention diagram describing the recruitment and randomization of participants in the study and the order in which the education tests and education materials were assigned for each group.

Pediatric patients (ages four to 15 years) and caregiver dyads were recruited from the university hospital within 48 hours of diagnosis, and then randomized using a web-based random number generator. Half (n=8) of the participants were randomized to NODE-enhanced DSMES intervention and the others (n=8) into the Standard DSMES control group (Figure 3). The Diabetes Knowledge Test 2 (DKT2) was used to assess preliminary efficacy. The DKT2 is a 23-item validated scoring instrument, developed for both type 1 and type 2 diabetes [6]. The DKT2 includes a 14-item general sub score, and a nine-item insulin-dependent sub score, with total scores ranging from 0 to 23. NODE usability was evaluated using the System Usability Survey (SUS), a 10-item general-purpose software usability scoring instrument, with scores ranging between 0 and 100 [7], [8]. Higher scores indicate higher levels of usability (>92 = best imaginable, >85 = excellent, >72 = good, >52 = average). For all participants, demographic information of the child was collected (sex, age, ethnicity, race, preferred language) and socio-economic status of the caregiver (income, years of education) as well as the family’s baseline DKT2 score. After completion of baseline measures, the intervention group received NODE, followed by standard DSMES with a certified diabetes educator in the hospital (if admitted at diagnosis) or clinic.

Following diabetes education which typically consists of three to four hours of one-on-one instruction within the first two days of diagnosis, participants completed the DKT2 and SUS. The control group received standard of care DSMES by a CDE, followed by administration of the DKT2. Parents completed all surveys in both the intervention and control groups immediately before any DSMES was initiated and the following day after their initial education was completed. Pre- and postintervention changes in DKT scores were assessed using T-tests as were intergroup differences. SUS scores were tallied for participants in the intervention group and descriptive statistics were generated (see Figure 3).

## Results

Sixteen child-caregiver dyads were recruited and randomized between July 2016 and January 2017. The ages of pediatric patients ranged from four to 15 years, with a mean age of 10.75 (SD 3.44). Demographics and socioeconomic status variables are summarized in Table 1.

**Figure 4:**
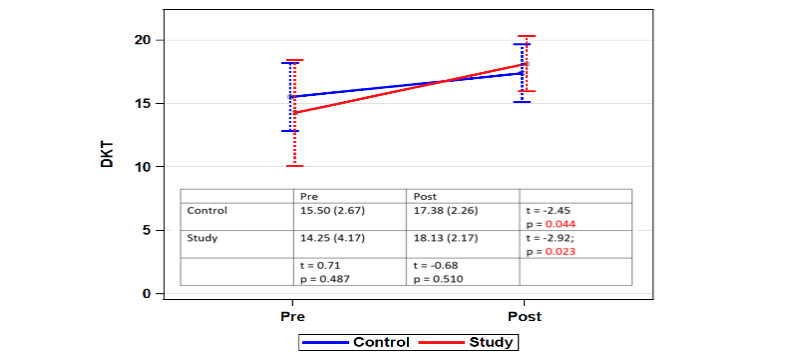
Diabetes Knowledge Test (DKT) score change across groups, describing the pre- and postintervention DKT2 scores for the group receiving standard nurse education versus the New-Onset Diabetes Educator (NODE) group.

At baseline, there was no significant difference in diabetes knowledge between groups (*t*=0.71, *P*=.487). DKT2 scores for the intervention group were 14.25 (SD 4.17) pretest and 18.13 (SD 2.17) posttest, demonstrating a statistically significant increase in knowledge acquisition (*t*=–2.492, *P*=0.023). DKT2 scores for the control group were 15.50 (SD 2.67) pretest and 17.38 (SD 2.26) posttest, also demonstrating a statistically significant increase in diabetes knowledge (*t*=–2.45, *P*=.044). These results are summarized in Figure 4. Overall, the effect size, defined as the difference between the standardized mean change for the intervention group and the control group was medium (Δ=0.56). The intervention group demonstrated more improvement in education acquisition than the control group (3.88 vs 1.88); however, this additional knowledge gain was not statistically significant (*t*=–1.30, *P*=.213). The SUS total mean score was 89.2 out of a 100 possible, representing excellent usability.

**Table 1:** Demographics and socioeconomic status. NODE: New-Onset Diabetes Educator.

Characteristics		Control group, n (%)	NODE group, n (%)
**Sex**			
	Female	6 (75)	4 (50)
	Male	2 (25)	4 (50)
**Race / Ethnicity**			
	White	6 (75)	7 (87.5)
	African-American or Mixed	2 (25)	1 (12.5)
	Hispanic / Latino	2 (25)	1 (12.5)
**Education**			
	High School or lower	5 (62.5)	4 (50)
	Associate degree or higher	3 (37.5)	4 (50)
**Income**			
	< $25,000	3 (37.5)	0 (0)
	$25,000 - $49,999	1 (12.5)	2 (25)
	$50,000 - $74,999	2 (25)	3 (37.5)
	$75,000 - $99,999	1 (12.5)	0 (0)
	> $100,000	1 (12.5)	1 (12.5)
	Not applicable / Refused to say	0 (0)	2 (25)
**Preferred Language**			
	English	8 (100)	8 (100)
	Spanish	0 (0%)	0 (0%)

## Discussion

In this study, we have demonstrated the usability of NODE in a university diabetes practice setting. Though there were no statistical differences between the intervention and the control group in terms of diabetes knowledge acquisition, the intervention group exhibited significant improvements in their DKT2 scores indicating non-inferiority of treatment and a lack of negative interference with the DSMES provided by the CDE. The diabetes educators did not communicate concerns regarding the use of NODE interfering with scheduling of their time with the patient and on a few occasions reflected that their educational interaction with the patient was improved by having an initial exposure to NODE. This benefit is important as NODE is meant to be used in conjunction with standard of care DSMES when a child is diagnosed with type 1 diabetes, rather than as a standalone iPad-based education platform.

NODE was deemed highly usable by the study participants. This provides preliminary evidence that the NODE web application is a feasible intervention to enhance diabetes education for child-caregiver dyads at the time of diagnosis. Finally, NODE can easily be used at home after being discharged from hospital, and therefore has the potential to greatly increase diabetes knowledge retention, and potentially, long-term clinical outcomes for patients with type 1 diabetes. In both groups, the parents completed the survey and knowledge test but often there was a group effort or discussion in replying to the questions which involved the child. The DKT2 is not designed to assess initial diabetes knowledge and has limited capacity to test the specific material information in NODE or in any new-onset education curriculum. A pediatric-specific scale should be used in future assessments to determine if diabetes knowledge acquisition is improved. A potential weakness of the study is the small sample size, which does not allow us to stratify across race and ethnicity. However, in the context of a pilot study, this is acceptable, and still allows us to demonstrate non-inferiority, and obtain preliminary effect size for a larger study.

Given the ubiquitous nature of mobile devices across the socio-economic status spectrum [9], and substantial evidence that mobile health can improve long term clinical outcomes [10] in particular among youth [11], NODE can be used later at home and thus facilitate not only diabetes-specific knowledge acquisition, but also retention.

Having demonstrated the feasibility of implementation in the clinical setting we will build on the goal of improving efficacy in acquisition of knowledge and retention.

## Abbreviations

DKT2: Diabetes Knowledge Test 2
DSMES: Diabetes Self-Management Education and Support
IRB: Institutional Review Board
NODE: New-Onset Diabetes Educator
SUS: System Usability Survey
